# P-925. Diagnostic value of 18-F-FDG-PET scan in endovascular infections

**DOI:** 10.1093/ofid/ofae631.1116

**Published:** 2025-01-29

**Authors:** Ajithkumar Ittaman, Hamad Abdel Hadi

**Affiliations:** HAMAD MEDICAL CORPORATION, doha, Ad Dawhah, Qatar; Communicable Diseases Centre, Hamad Medical Corporation, Doha, Ad Dawhah, Qatar

## Abstract

**Background:**

There have been multiple studies evaluating the use of 18-fluorine-fluorodeoxyglucose positron emission tomography (18F-FDG PET) as a diagnostic tool for endovascular infections with mixed results. The aim of this study will be to assess the efficacy of 18F-FDG PET scan in endovascular infections

demographic details of confirmed IE by criteria

**Figure:**
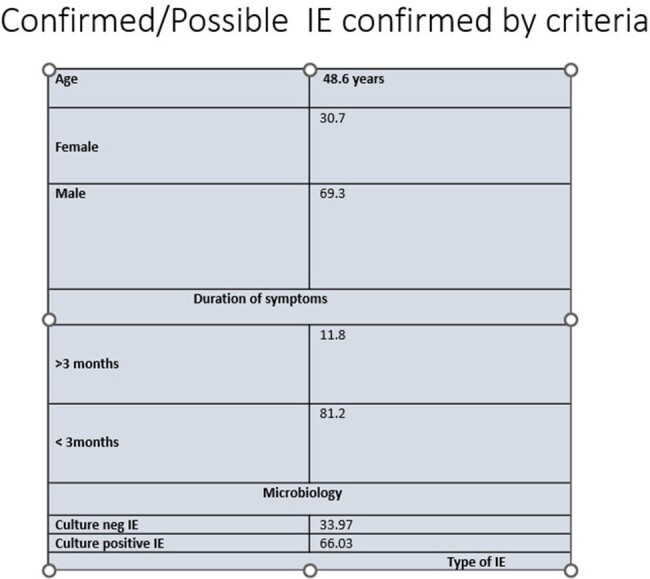

**Methods:**

Our study plan to evaluate the accuracy of PET scan in diagnosing VPGI, infective endocarditis and other endovascular infection. We will use MAGIC criteria^3^ and Modified Dukes criteria ^1^ regarding comparative diagnosing evaluation of VPGI and Infective endocarditis which has been previously validated. It will be correlated with 18 FDG PET report to calculate sensitivity, specificity, negative and positive predictive value of PET scan in diagnosing endovascular infections.

IE according to location (True positives- as per criteria )

**Figure:**
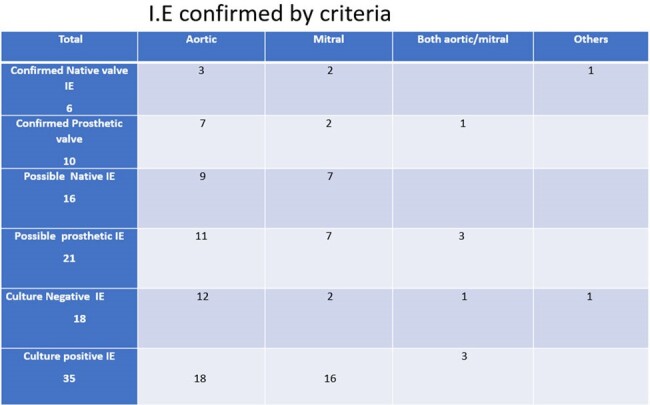

**Results:**

•Referred with suspected IE ,Graft infections, endovascular infections and FUO- 158 cases.Analysed PET scan report – IE ,endovascular infections.

PET scan with positive/negative reports for endovascular infections was analysed with confirmed cases by Modified Dukes and MAGIC criteria to calculate sensitivity, specificity, positive and negative predictive value.

- 53- Confirmed and possible IE.Of 53 , 44 positive in PET scan Sensitivity- 83.01%.
  - PET scan done for 14 suspected cases Vascular graft infections. Sensitivity of 64.28%.
    - Sensitivity is variable: excellent for the diagnosis of PVE and CDRIE-pocket infections, but poor for NVE and CDRIE-lead infections.

**Conclusion:**

PET scan was found to be efficient diagnostic tool in endocarditis and vascular graft infection.Need prospective studies for validation in vascular graft inefctions

**Disclosures:**

**All Authors**: No reported disclosures

